# Teletherapy sources with imported and indigenous ^60^Co activity

**DOI:** 10.4103/0971-6203.54854

**Published:** 2009

**Authors:** Jain Reji George, Raksha Kushwah, K. V. S. Sastry

**Affiliations:** Board of Radiation and Isotope Technology, Department of Atomic Energy, BRIT/BARC Vashi Complex, Sector-20, Vashi, Navi Mumbai-400705, India

**Keywords:** Content activity and specific activity, radiation output (RMM), teletherapy, ^60^Co pellets

## Abstract

Board of Radiation and Isotope Technology, a unit of the Department of Atomic Energy, fabricates and supplies radioactive sources for medical, industrial, agriculture and research applications. High specific activity cobalt-60, required for teletherapy is normally imported. There was a proposal for manufacturing high specific activity sources indigenously. A study was carried out to observe the feasibility of mixing imported and indigenous cobalt-60 pellets to fabricate teletherapy source capsules. The specific activity of imported pellets is more than 300 Ci/g, whereas that of indigenous pellets obtained from Indian power reactors is 140 Ci/g. The radiation output from a capsule for different combinations of specific activity was evaluated. Losses due to self-absorption were accounted in the evaluations. In another study, the optimized lengths of the capsule for an output of 200 RMM and the additional activity to be added to compensate losses due to self-absorption were also estimated for different specific activity pellets. Sources fabricated on the basis of this study showed a good agreement with the estimations. Source capsules with a combination of different specific activities are yet to be fabricated.

## Introduction

Radiotherapy is a clinical specialty in which ionizing radiations are used to treat patients with malignant neoplasm. Radiotherapy can be either delivered as teletherapy (from a distance) or brachytherapy (from near).[1,2] In teletherapy, the source is external to body normally housed in a well-shielded containment known as teletherapy machine. The teletherapy machine using isotope had begun with radium as the source. However, at present the source in a teletherapy machine is cobalt-60 (^60^Co). The Board of Radiation & Isotope Technology, a unit of the Department of Atomic Energy, fabricates and supplies radioactive sources for medical, industrial, agriculture and research applications. High specific activity (up to 320 Ci/g) ^60^Co pellets required for teletherapy is normally imported. There was a proposal of indigenously manufacturing sources of specific activity of 160 to 180 Ci/g. ^60^Co required for the fabrication of sources is produced in nuclear power reactors by the following nuclear reaction: ^59^Co (n,γ) →^60^Co. Cobalt metal (^59^Co) is loaded in adjustor rods in the form of nickel-plated cobalt pellets with the following dimensions: 1 mm diameter and 1 mm height. The activity induced depends on the duration of exposure to the neutron flux in the reactor. The adjustor rods from the power reactors are then transported to the cobalt handling facility and then cut under water. The subassemblies are taken to hot cells for recovery of activity, which are used for fabrication of sources. This article presents the feasibility study to mix the imported and indigenous ^60^Co pellets to fabricate teletherapy source capsules. The study dealt with the total activity to be filled, self-absorption losses and the radiation output of the source capsules.

## Materials and Methods

The specifications of the source capsule and pellets considered were as follows:

**Table:** 

Internal diameter of source capsule:	20 mm
Length of source capsule:	29 mm
Size of ^60^Co pellets:	1 mm × 1 mm
Specific activity of imported pellets:	320 Ci/g
Specific activity of indigenous pellets:	140 Ci/g
Packing density of pellets in the capsule:	5.4 g/cc

**Figure 1:**
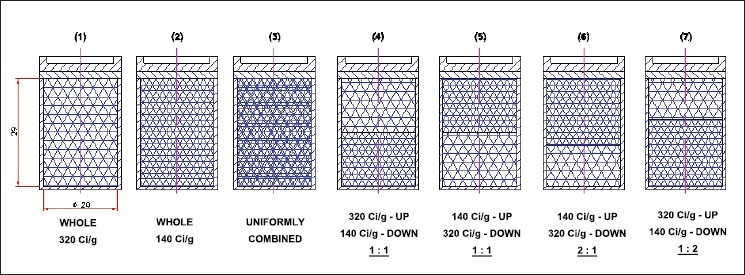
Telecobalt source capsule

At first, the source capsule was assumed to be filled with imported and indigenous specific activity ^60^Co pellets separately and then with a combination of the imported and indigenous pellets in different (known) ratios. The following cases were studied when the source capsule [Figure 1] was filled with materials as given here:

1. Only imported specific activity (320 Ci/g) [Figure 1] – (1)
2. Only indigenous specific activity (140 Ci/g) [Figure 1] – (2)
3. Uniformly with both imported and indigenous [Figure 1] – (3)
4. Top with 320 Ci/g and bottom with 140 Ci/g in 1:1 ratio [Figure 1] – (4)
5. Top with 140 Ci/g and bottom with 320 Ci/g in 1:1 ratio [Figure 1] – (5)
6. Top with 140 Ci/g and bottom with 320 Ci/g in 2:1 ratio [Figure 1] – (6)
7. Top with 320 Ci/g and bottom with 140 Ci/g in 1:2 ratio [Figure 1] – (7)

The radiation outputs for the different combinations were evaluated as follows. By knowing the mass of the pellets of different specific activities, the content activity in the source capsule was determined. Assuming the part of the capsule with the same specific activity as a sphere of radius R, the corresponding self-absorption distance Z was determined.[3,4] The self-absorption distance is an equivalent distance for R. From this the effective self-absorption factor = e^−μZ^ and the effective activity = content activity X e^−μZ^ were determined, where *μ* is the linear attenuation coefficient of the source material. The radiation output was determined as specific gamma ray constant X effective activity/distance^2^. The output was represented in RMM,[5] which is the radiation exposure in R/min at 1 m from the source.

In another part of the study, the active length of the source capsule was optimized for a target output of 200 RMM. The active lengths were evaluated for three different specific activities, 250, 270 and 300 Ci/g. From the effective activity in a capsule (which is the same for all), the mass of the capsule and lengths were calculated for a known packing density of the pellets. The self-absorption losses for them were evaluated as explained above and the additional activity to be filled for compensating the losses was determined.

## Discussion on the Results and Conclusion

The results of the first part of the study are given in Table 1. The radiation output from the source capsule for RMM was evaluated in this study. Among the combinations of different specific activity pellets, the combination with 140 Ci/g up and 320 Ci/g down in 1:1 ratio (number 5) yields an output of 196.8. Even the combination numbers 3, 4 and 6 yield an RMM greater than 170, which also can be considered for fabrication. The specific activity of indigenous pellets obtained from Indian power reactors reached up to 160 to 180 Ci/g. It may increase in future depending on the operation of the reactor. Thus, the study shows that indigenous pellets can be used for fabrication of teletherapy source capsules, in combination with the imported pellets to give the radiation output necessary for treatment. Table 2 shows the results of the second part of the study. The source capsules fabricated on the basis of this analysis showed losses due to self-absorption and the additional activity added to compensate the losses were as estimated.

**Table 1:** Radiation output from source capsule

*Specific activity of ^60^Co pellets*		*Content activity (Ci)*	*RMM*
1.	Only 320 Ci/g	14400	250.3
2.	Only 140 Ci/g	6300	109.5
3.	320 and 140 Ci/g-uniformly combined	10350	179.9
4.	320 Ci/g up and	7200	178.2
	140 Ci/g down (1:1)	3150	
5.	140 Ci/g up and	3150	196.8
	320 Ci/g down (1:1)	7200	
6.	140 Ci/g up and	4200	170.9
	320 Ci/g down (2:1)	4800	
7.	320 Ci/g up and	4800	152
	140 Ci/g down (1:2)	4200	

**Table 2:** Optimization of source capsule length

*Specific activity (Ci/g) (mm)*	*Content activity (Ci)*	*% Loss due to self-absorption*	*Active length of capsule*
250	11455	26.0%	21.4
270	11318	24.5%	19.8
300	11036	21.4%	17.8
